# Pre–treatment method to avoid contamination for radiocarbon dating of organic–rich coastal deposits

**DOI:** 10.1016/j.mex.2022.101745

**Published:** 2022-05-28

**Authors:** Qiang Yao, Kam-biu Liu, Erika Rodrigues

**Affiliations:** Department of Oceanography and Coastal Sciences, College of the Coast and Environment, Louisiana State University, Baton Rouge, LA 70803, USA

**Keywords:** radiocarbon dating, coastal deposits, paleoecology, organic sediments

## Abstract

Establishing an accurate chronology for coastal sediment profiles using radiocarbon dating has been a challenging task for scientists around the world. In this study, we present a step-by-step procedure of an optimized pre-treatment method to remove roots, shell hashes, and other contaminants from organic-rich bulk sediments for radiocarbon dating. This procedure first applies loss-on-ignition analysis throughout the sediment profile to locate the ideal sampling intervals that have high organic and low carbonate contents, and then uses a two-step sieving procedure to remove contaminants from the bulk sediments. During the past five years, we have prepared a total of 64 samples for radiocarbon dating using this pre-treatment method, and 59 of them were deemed valid, a success rate of 92.2%. Thus, we believe this procedure can successfully remove contamination and optimize the sample pre-treatment for radiocarbon dating of organic-rich deposits from coastal and other environments.

• Use loss-on-ignition analysis to locate the ideal sampling intervals.
• Sieve each sample with 200 µm and then 100 µm sieve to remove roots and organic debris.
• Acid-leach each of the sieved sediment samples (100-200 µm) with HCL to remove carbonates.

## Graphical abstract

**Figure:**
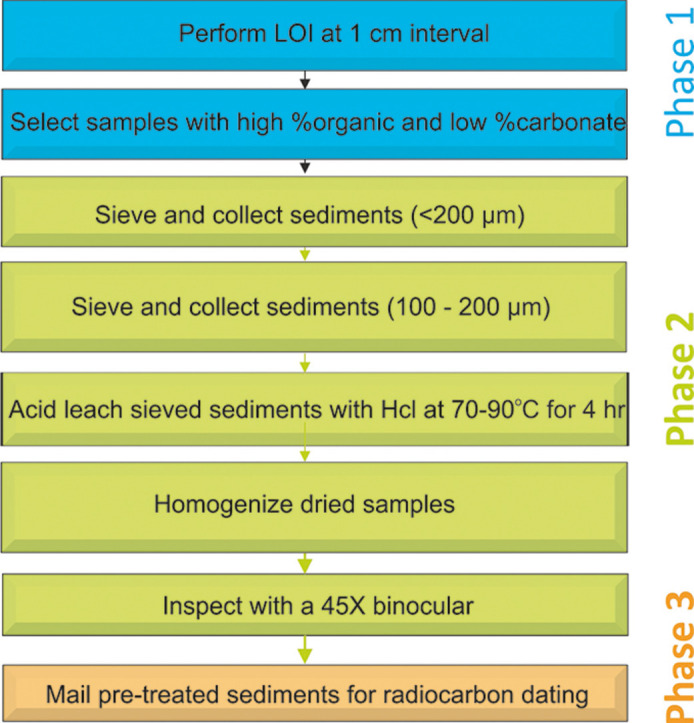

Specifications table

**Table:** 

Subject Area;	Earth and Planetary Sciences
More specific subject area;	*Paleoecology*
Method name;	*Pre-treatment for radiocarbon dating*
Name and reference of original method;	*N/A*
Resource availability;	*N/A*

## METHOD DETAILS

### Rationale

Coastal settings are highly dynamic and complex environments that frequently encounter natural and anthropogenic disturbances. On the shorter timescales, events like storms and seismic activities can significantly disturb the stratigraphy of coastal sediment profiles. On timescales of centuries to millennia, sea-level fluctuations, subsidence, and climate anomalies (e.g., the “Medieval Warm Period” and “Little Ice Age”) can also have profound impact on the sediment accretion rate. Thus, establishing an accurate chronology for coastal sediment profiles has been a challenging task for scientists around the world.

To date, radiocarbon dating is still the most common method to establish chronology for coastal deposits in palaeoecological research. This method is time-tested and relies on the properties of radioactive isotope of carbon in organic materials [2]. However, because of its dependence on organic materials, several factors can cause contamination and affect the accuracy of the radiocarbon dating results, particularly when dating bulk sediments. One major problem is that due to the dynamic nature of the coastal environments, “old” materials (e.g., driftwood and shell) can be introduced to the sediment profile and result in anomalously old dates. Another common problem is that plant roots can penetrate and contaminate deeper sediment profiles and cause anomalously young dates. In this study, we present a step-by-step procedure of an optimized pre-treatment method and some insights to avoid contamination by shell harsh, driftwood, and rootlets for radiocarbon dating of bulk organic-rich deposits.

### Required materials and instruments

#### General materials

1. Nitrile Exam Gloves
2. Weigh boat
3. Measuring spoon
4. Electronic balance (Fig. 1a)

**Fig. 1:**
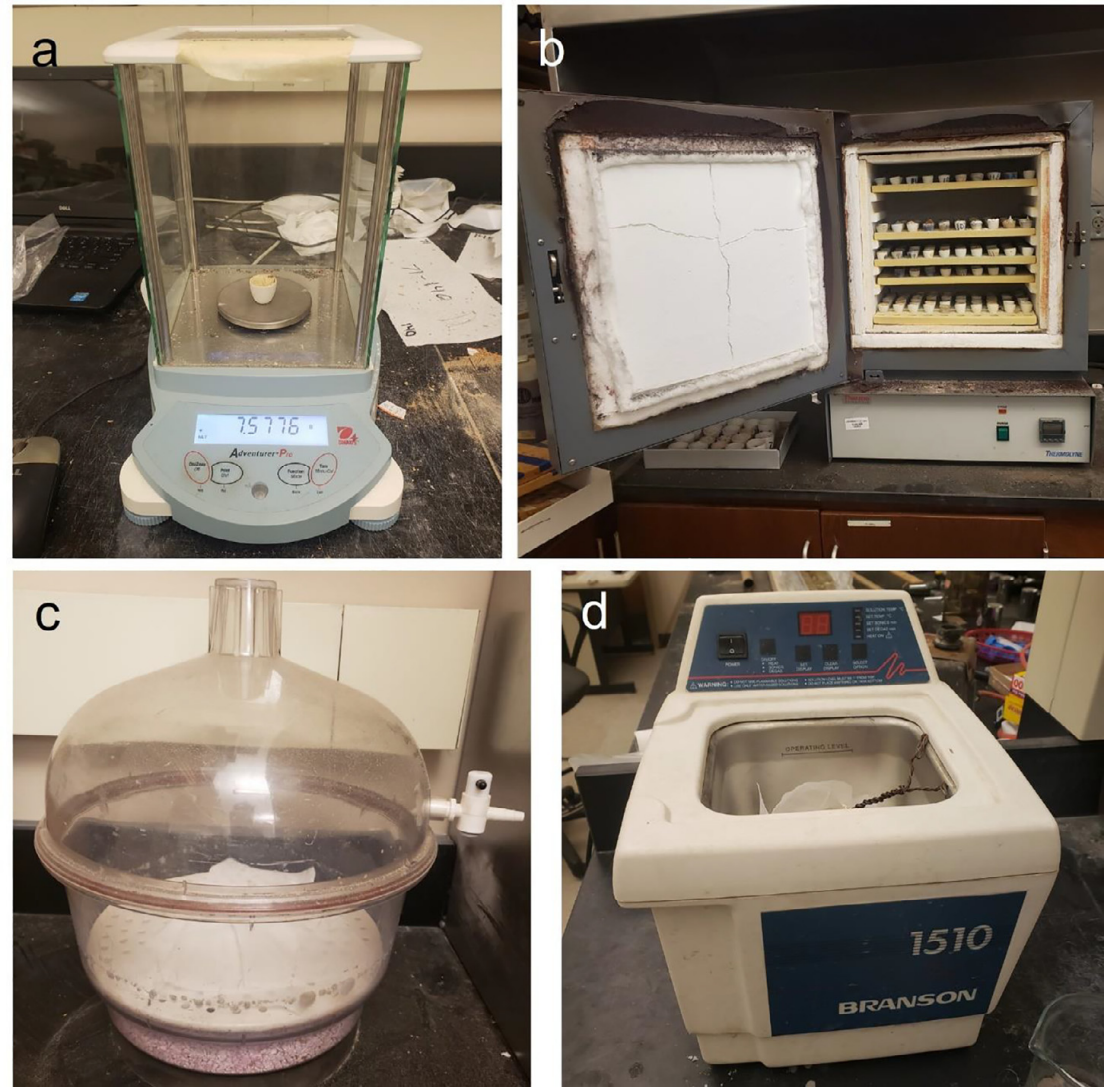
Essential instruments for radiocarbon dating pre-treatment, such as electronic balance (a), muffle furnace and ceramic crucible (b), desiccator (c), and sonicator (d)
5. Deionized water
6. Muffle furnace (Fig. 1b)

#### Loss-on-Ignition

1. Desiccator (Fig. 1c)
2. Ceramic crucible (Fig. 1b)
3. Porcelain crucible marking ink

#### Sediment sieving

1. 200 µm nylon or metal mesh sieve
2. 100 µm nylon or metal mesh sieve
3. Hydrochloric acid (0.5 to 1.0 N)
4. Mortar and pestle
5. Binocular microscope
6. Sonicator (Fig. 1d)

### Procedures

#### Locate the ideal sample interval with Loss-on-Ignition

1. Mark the crucibles in alphabetical or numerical order.
2. Weigh and record each of the marked crucibles with an electronic balance.
3. Add 2 full spoons (3-5g) of sediments to each crucible at 1 cm interval throughout the sediment profile (or sediment cores in most cases).
4. Put samples in the muffle furnace at 105°C to dry overnight (8 -12 hr).
5. Weigh and record each of the crucibles with dry sediments.
6. Put samples in the muffle furnace and wait till it reaches 550°C then heat for 1 hour.
7. Weigh and record each of the crucibles and place them in a desiccator
8. Use this formula to calculate the % organic (dry)

(weight_step5_ - weight_step7_) / (weight_step5_ - weight_step2_) * 100%

9. Put samples in the muffle furnace and wait till it reaches 1000°C then heat for 1 hour.
10. Weigh and record each of the crucibles.
11. Use this formula to calculate the % carbonate (dry)

(weight_step7_ - weight_step10_) / (weight_step7_ - weight_step2_) * 100%

**Fig. 2:**
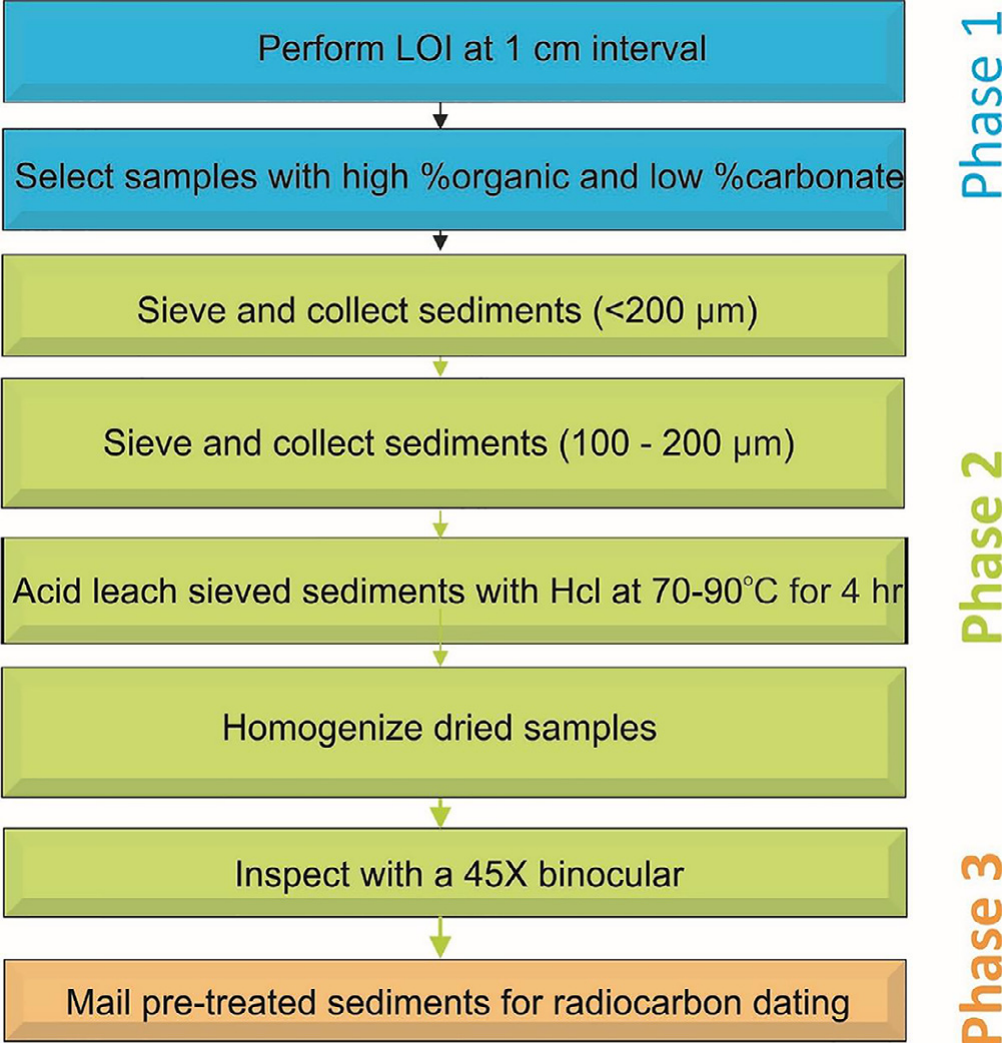
Methodology flow-chart

12. Select intervals with high organic content and low carbonate content from the sediment profile/core for radiocarbon dating Fig. 2.

#### Prepare sediment samples for radiocarbon dating

1. Disperse each sample in deionized water.
2. Sieve each sample with 200 µm sieve and collect the fine fraction (<200 µm) of the sieved sediments (Fig. 3).

**Fig. 3:**
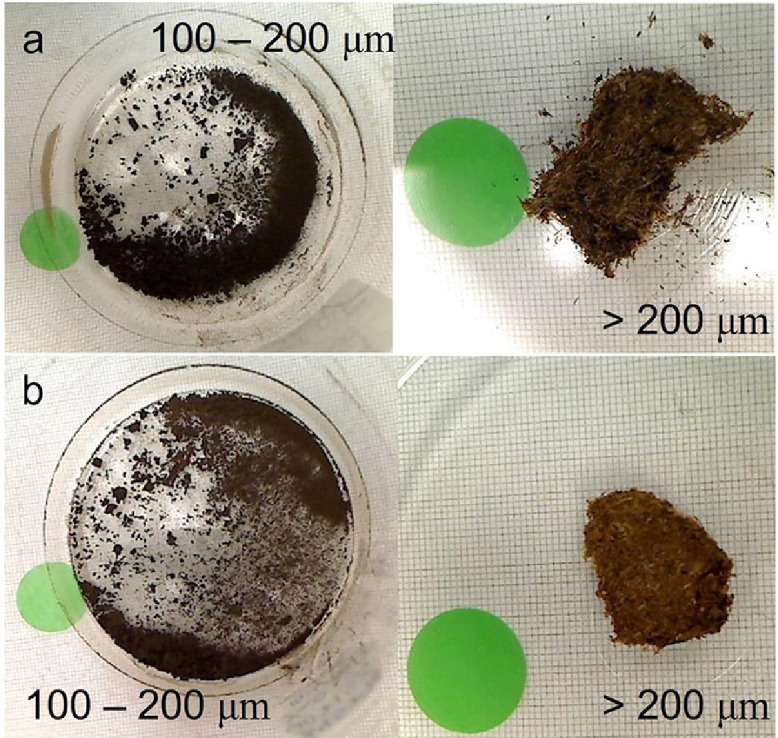
Two pairs of samples showing the fine (100-200 µm) and coarse (>200 µm) fractions of the sieved sediments. The green dot is 24 mm in diameter and shown as scale.
3. Sieve each sample with 100 µm sieve and collect the coarse fraction (100-200 µm) of the sieved sediments (Fig. 3). Due to the small mesh size, use a sonicator to aid finer grain materials (<100 µm) to pass through the sieve.
4. Acid leach sieved sediment samples (100-200 µm) with Hydrochloric Acid solution (0.5 to 1.0 N) at near boiling temperatures (70-90°C) for 4 hr to remove carbonates.
5. Dry each sample for 8-12 hr in an oven at 70°C.
6. Inspect under a 45 × binocular microscope to remove the remaining shell fragments and fibrous tissues.
7. Homogenize each of the dried samples with mortar and pestle
8. Put each of the pre-treated samples in a secure packing and mail the packaged samples for radiocarbon dating.

## Method validation

In general, this pre-treatment procedure can remove driftwoods, shell hashes, and other

“old” carbonate and “young” plant fibers, particularly rootlets or root hairs that intruded downward into deeper sediment profiles (Fig. 3). This pre-treatment procedure will also separate samples into two fractions, a fine fraction (100-200 µm) containing decomposed plant materials and a coarse fraction containing predominantly elongated plant fibers that resembled rootlets or root hairs (>200 µm). It is reasonable to infer that the fine fraction (100-200 µm) sediments represent the deposed *in-situ* plant detritus without roots, hence the ideal material for radiocarbon dating.

During the past five years, we have published a total of 46 radiocarbon dates using this pre-treatment method, and 42 of them match the age–depth model developed by BACON version 2.2 (a Bayesian age–depth modeling software using R as an interface) [1], hence, a success rate of 91.3% (Table. 1). If unpublished data are included, we have prepared a total of 64 samples for radiocarbon dating using this pre-treatment method, and 59 of the dates were deemed valid, a success rate of 92.2%. Thus, we believe this procedure removes contamination and optimizes the sample pre-treatment for radiocarbon dating of organic-rich bulk deposits.

**Table 1:** Radiocarbon dating results from our published articles during the past 5 years [3], [4], [5], [6]. Gray color marks the samples that did not use the pre-treatment method described in this study.

CORE ID	SAMPLEDEPTH (CM)	CONVENTIONAL AGE (BP)	2-SIGMA CALIBRATEDAGE (cal yr BP)	REFERENCE
BBS-1	32	modern	modern	[6]
BBS-1	127	440 +/- 30	490	
BC-53	80	795 +/- 100	740	
BC-53	115	1720 +/- 20	1610	
BC-53	210	4220 +/- 20	4760	
SGI-3	50	660 +/- 40	620	
SGI-3	100	690 +/- 30	670	
SGI-3	215	1630 +/- 30	1490	
SGI-4	25	modern	modern	
SGI-4	80	740 +/- 30	680	
SGI-5	39	modern	modern	
LAG-3	65	361 +/- 23	420	
LAG-3	95	960 +/- 26	860	
LAG-4	25	modern	modern	
LAG-4	65	589 +/- 23	610	
LAG-5	55	modern	modern	
LAG-5	95	1019 +/- 24	940	
LAG-6	40	51 +/- 23	0	
ROC-4	78	580 ± 30 BP	590	[4]
ROC-4	403	3900 ± 30 BP	4340	
ROC-4	525	4530 ± 30 BP	5190	
SRM	56	145 ±25	140	[3]
SRM	139	1180 ±30	1085	
SRM	179	1940 ±30	1895	
SRM	243	2860 ±30	2975	
SRM	260	2240 ±30	rejected	
SRM	374	4160 ±30	4805	
SRM	440	1090 ±20	rejected	
SRM	446	5800 ±30	6585	
SRS-6	200	2260 ±20	2250	
SRS-6	232	2970 ±90	3140	
SRS-6	303	3570 ±25	3870	
SRS-6	378	4230 ±40	4745	
SRS-5	155	2050 ±25	2020	
SRS-5	190	2560 ±20	2645	
SRS-5	248	4010 ±25	4470	
SRS-4	90	1180 ±30	1105	
SRS-4	100	1530 ±20	1440	
SRS-4	125	2460 ±20	2550	
Cocos-7	17	modern	modern	[5]
Cocos-7	25	990 +/- 40	rejected	
Cocos-7	40	370 +/- 30	430	
Cocos-7	55	500 +/- 30	530	
Cocos-7	60	1150 +/- 40	rejected	
Cocos-7	72	740 +/- 30	680	
Cocos-7	79	760 +/- 50	700	
Cocos-9	35	1020 +/- 40	rejected	
Cocos-11	20	1680 +/- 40 BP	rejected	
Cocos-11	45	860 +/- 30	770	

## Conclusion

In this study, we present an optimized pre-treatment method that can remove contamination and increase accuracy for radiocarbon dating of organic-rich deposits from coastal and other environments. More importantly, this method produces consistent bulk materials for radiocarbon dating, instead of “eye-balling” organic materials (e.g., leaf and bark) that solely relies on the empirical experience of the operator. We want to point out that the loss-on-ignition is an optional step to locate the ideal stratigraphic positions in the sediment profile to be sampled for dating. The burned sediments should be decanted, and this step can be omitted if visual inspection reveals that the entire sediment profile has high-organic and low-carbonate contents. In addition, although this pre-treatment method can be applied to low organic sediments, the optimal materials are sediments with >10% organic content. Last but not the least, the mesh size for the sieve (100 - 200 µm) is a general guideline, the operator should feel free to choose the ideal size sieve for their samples.

## Declaration of Competing Interest

The authors declare that they have no known competing financial interests or personal relationships that could have appeared to influence the work reported in this paper.
